# Sulfonylurea for the treatment of neonatal diabetes owing to K_ATP_-channel mutations: a systematic review and meta-analysis

**DOI:** 10.18632/oncotarget.22548

**Published:** 2017-11-20

**Authors:** Hongliang Zhang, Xiaobin Zhong, Zhenguang Huang, Chun Huang, Taotao Liu, Yue Qiu

**Affiliations:** ^1^ Pharmacy Department, The First Affiliated Hospital of Guangxi Medical University, 530021, Nanning, China; ^2^ Guangxi Key Laboratory of Regenerative Medicine, The First Affiliated Hospital of Guangxi Medical University, 530021, Nanning, China

**Keywords:** sulfonylurea, neonatal diabetes, systematic review, meta-analysis

## Abstract

The effect of sulfonylurea for the treatment of neonatal diabetes (NDM) is remain uncertain. We conducted this systematic review and meta-analysis to investigate the effect of sulfonylurea for NDM and to provide the latest and most convincing evidence for developing clinical practice guidelines of NDM. A literature review was performed to identify all published studies reporting the sulfonylurea on the treatment of neonatal diabetes. The search included the following databases: PUBMED, EMBASE and the Cochrane Library. The primary outcome was the success rates of treatment, change of glycosylated hemoglobin (HbA1c) and C-peptide. Data results were pooled by using MetaAnalyst with a random-effects model. Ten studies (6 cohort studies and 4 cross-sectional studies) involving 285 participants were included in the analysis. The pooled estimated success rate by the random-effects model was 90.1%(95% CI: 85.1%–93.5%). HbA1c had a significantly lower compared with before treatment. The pooled estimate of MD was -2.289, and the 95% CI was -2.790 to -1.789 (*P* < 0.001). The subgroup analysis showed a similar result for cohort studies and in cross-sectional studies. The common mild side effect is gastrointestinal reaction. The present meta-analysis suggested that sulfonylurea had a positive effect for treatment NDM due to K_ATP_ channel mutations. In addition, sulfonylurea also displayed sound safety except the mild gastrointestinal reaction. However, the findings rely chiefly on data from observational studies. Further well-conducted trials are required to assess sulfonylurea for NDM.

## INTRODUCTION

Neonatal diabetes mellitus (NDM) is rare and estimated incidence is about 1 in 90,000–260,000 live births [1–3]. NDM defined as the occurrence of diabetes in the first 6 months of life [4]. It can be divided into two clinical subtypes: permanent neonatal diabetes mellitus (PNDM) that requires continuous treatment since diagnosis, and transient neonatal diabetes mellitus (TNDM) that typically resolves after a few weeks to months, but relapsing around puberty after a period of remission [5].

For a long period, the cause of NDM was unknown. Insulin treatment is generally acutely required in most infants with newly diagnosed diabetes mellitus to treat or prevent ketoacidosis and dehydration [6]. However, insulin therapy presents a particular challenge in these very young children with respect to compliance, and precise dosage.

Recently, activating mutations in the Kir6.2 and sulfonylurea receptor 1 (SUR1) subunits of the pancreatic ATP sensitive K_ATP_ channel, coded for by the genes KCNJ11 and ABCC8, have been identified major causes of NDM [7, 8]. Based on this key breakthrough, sulfonylurea which is widely used to treat type 2 diabetes, is becoming a new treatment option for NDM.

Sulfonylurea binds specifically to the SUR1 subunit, closing the K_ATP_ channel via an ATP-independent mechanism and therefore increasing the insulin secretion from the β cells [9]. However, sulfonylureas are not approved for use in infants in most countries. All guidelines and recommendations have also not mentioned the sulfonylureas for NDM.

Though the studies about sulfonylureas for activating mutations NDM have increased rapidly in the last few years [10, 11], due to small sample sizes, these studies were not adequately powered to detect the effect of sulfonylureas in NDM.

Therefore, we performed this systematic review and meta-analysis to investigate the effect of sulfonylurea for NDM. Furthermore, we provide the latest and most convincing evidence for developing clinical practice guidelines of NDM by this meta-analysis.

## RESULTS

### Study identification and selection

**Figure 1:**
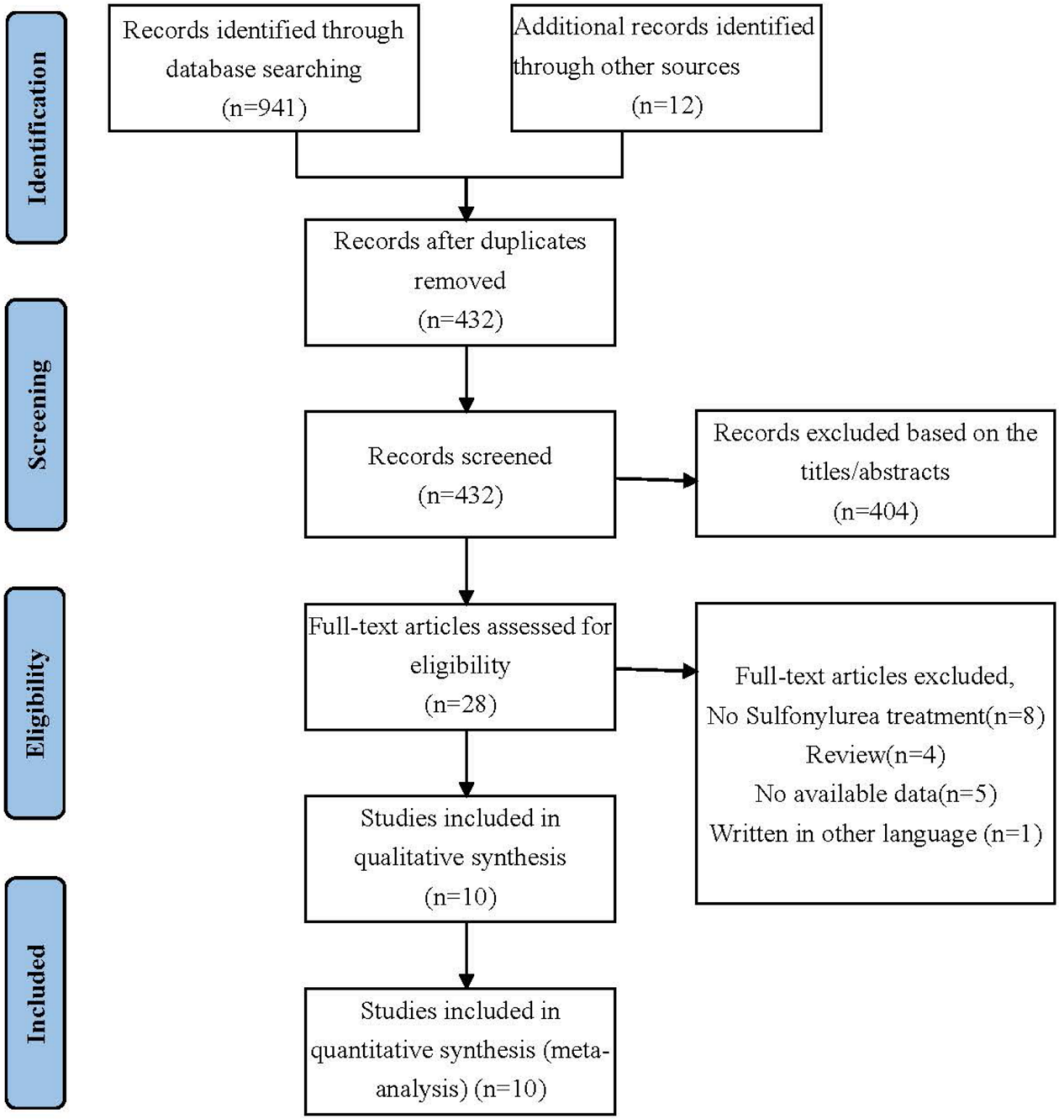
Selection process for the studies included in the meta-analysis

Initially, 941 records were retrieved from the database search and 12 additional records identified through other sources. After removing duplicate articles, 432 records were eligible. Based on the inclusion and exclusion criteria, 404 articles were excluded after a simple reading of the titles and abstracts of the articles. The remaining 28 full-text articles were assessed for eligibility. Then, no sulfonylurea treatment, review, no available data, written in other language were excluded. Finally, a total of 10 studies were included in the meta-analysis. The selection process is shown in Figure 1.

### Study characteristics

The main characteristics of the included studies are summarized in Table 1, and the outcome data of each included study are shown in Table 2. These studies were published from 2006 to 2016. The number of participants in the studies involving 285 participants ranged from 5 to 73. Six of 10 were cohort studies [9, 12–16], and 4 of 10 were cross-sectional studies [17–20].

**Table 1:** Search strategy

**Source: PubMed; Searched on: Jan 5, 2017**		
**Search**	**Query**	**Items found**
#11	Search #10 AND #3	395
#10	Search #6 AND #9	14766
#9	Search #7 OR #8	520111
#8	Search Diabetes [Title/Abstract]	411384
#7	Search “Diabetes Mellitus” [Mesh]	353111
#6	Search #4 OR #5	667779
#5	Search (neonatal [Title/Abstract]) OR Newborn [Title/Abstract]	273607
#4	Search “Infant, Newborn” [Mesh]	539228
#3	Search #1 OR #2	22050
#2	Search (((((((((glimepiride [Title/Abstract]) OR Acetohexamide [Title/Abstract]) OR Carbutamide [Title/Abstract]) OR Chlorpropamide [Title/Abstract]) OR Gliclazide[Title/Abstract]) OR Glipizide [Title/Abstract]) OR Glyburide [Title/Abstract]) OR Tolazamide [Title/Abstract]) OR Tolbutamide [Title/Abstract]) OR Sulfonylurea [Title/Abstract]	13505
#1	Search “Sulfonylurea Compounds” [Mesh]	17797
**Source: Embase; Searched on: Jan 5, 2017**		
**Search**	**Query**	**Items found**
#10	#3 AND #6 AND #9	556
#9	#7 OR #8	849821
#8	diabetes:ab,ti	586257
#7	‘diabetes mellitus’/exp	761547
#6	#4 OR #5	661321
#5	neonatal:ab,ti OR newborn:ab,ti	322871
#4	‘newborn’/exp	518403
#3	#1 OR #2	26280
#2	sulfonylurea:ab, ti OR glyburide:ab,ti OR glipizide:ab, ti OR gliclazide:ab, ti OR glimepiride:ab, ti OR acetohexamide:ab, ti OR carbutamide:ab, ti ORchlorpropamide:ab, ti OR tolazamide:ab, ti OR tolbutamide:ab, ti	17822
#1	‘sulfonylurea’/exp	11821

**Table 2:** Characteristics of included studies

Study	Country	Study design	Participants (KCNJ11/ABCC8 mutant)	Transfer sulfonylurea therapy	Successful treatment	Treatment regimen	Treatment time	Outcome indicators
Ewan R. Pearson 2006 [17]	UK	cohort study	49	49	44	glibenclamide	12 weeks	HbA1c
Juraj Stanik 2007 [24]	Slovakia	cross-sectional study	5	5	4	glibenclamide	1 month; 6 months	HbA1c, CGMS, C-peptide
Meena Rafiq 2008 [18]	UK	cohort study	27	27	23	glibenclamide		HbA1c
Jahnavi S 2013 [23]	India	cross-sectional study	10	5	5	glibenclamide		HbA1c, glucose tolerant
David Carmody 2014 [21]	USA	cross-sectional study	73	73	69	glibenclamide		
Brian W. Thurber 2015 [20]	USA	cohort study	58	58	58	glibenclamide		HbA1c
Jacques Beltrand 2015 [16]	France	cohort study	18	18	18	glibenclamide		HbA1c, C-peptide
Evgenia Globa 2015 [22]	Ukraine	cross-sectional study	12	12	11	glibenclamide	3 months; 1 year	HbA1c
Patricia Taberner 2016 [19]	Argentina	Wcohort study	8	5	4	glibenclamide	3 months	HbA1c, C-peptide
Yukiko Hashimoto 2016 [9]	Japan	cohort study	25	17	14	glibenclamide		HbA1c, C-peptide

### Risk of bias assessment

Risk of bias assessment of the included studies is summarized in Tables 3 and 4. Based on the Newcastle-Ottawa Scale (NOS) to assess the risk of bias of the cohort studies, 5 studies [9, 12, 13, 15, 16] were rated as a total score of 9 and one study [14] scored 8, indicating a low risk of bias. According to the Agency for Healthcare Research and Quality (AHRQ) items to assess the cross-sectional studies, all four studies [17–20] are categorized moderate quality.

**Table 3:** Outcome data of included studies

Study	HbA1c		Basal C-peptide, ng/mL	
	Before treatment	After treatment	Before treatment	After treatment
Ewan R. Pearson 2006 [17]	8.1 (7.7–8.6)	6.4 (6.2–6.6)	–	–
Juraj Stanik 2007 [24]	10.0 ± 0.73	6.2 ± 0.37	0.04 ± 0.04	0.73 ± 0.07
Meena Rafiq 2008 [18]	7.2 (6.6–8.2)	5.5 (5.3–6.2)	–	–
Jahnavi S 2013 [23]	10.18 ± 2.6	6.84 ± 0.46	–	–
David Carmody 2014 [21]	–	–	–	–
Brian W. Thurber 2015 [20]	8.5 ± 1.8	6.2 ± 1.0	–	–
Jacques Beltrand 2015 [16]	7.75 (5.5–12.8)	6.4 (5.4–10)	0.07 (0.02–0.51)	0.28 (0.12–0.82)
Evgenia Globa 2015 [22]	7.4 (6.6–9.6)	5.6 (5.4–5.9)	–	–
Patricia Taberner 2016 [19]	8.56 ± 0.56	5.80 ± 1.07	0.15 ± 0.10	1.29 ± 0.97
Yukiko Hashimoto 2016 [9]	–	6.4(4.9–8.5)	–	–

**Table 4:** NOS quality assessment of included cohort studies

Items	Study	Ewan R. Pearson 2006	Meena Rafiq 2008	Brian W. Thurber 2015	Jacques Beltrand 2015	Patricia Taberner 2016	Yukiko Hashimoto 2016
Selection	Representativeness of the exposed cohort	★	★	★	★	★	★
	Selection of the non-exposed cohort	★	★	★	★	★	★
	Ascertainment of exposure	★	★	★	★	★	★
	Demonstration that outcome of interest was not present at start of study	★	★	★	★	★	★
Comparability	Comparability of cohorts on the basis of the design or analysis	★★	★★	★★	★★	★★	★★
Outcome	Assessment of outcome	★	★	★	★	★	★
	Was follow-up long enough for outcomes to occur	★	–	★	★	★	★
	Adequacy of follow up of cohorts	★	★	★	★	★	★
**Quality Scores**		9	8	9	9	9	9

### Treatment success rate

**Figure 2:**
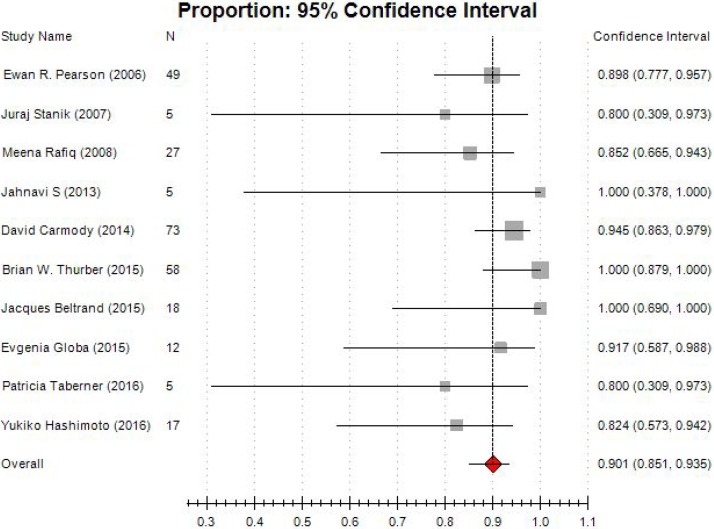
Forest plot of meta-analysis on treatment success rate

**Figure 3:**
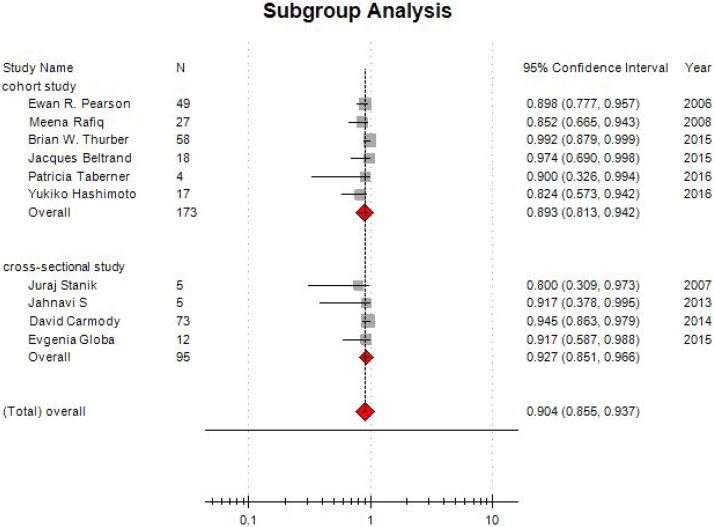
Funnel plot of subgroup analysis on treatment success rate

In all studies, treatment success rate was varying from 80% to 100%. Low heterogeneity (I^2^=0.00, *P* = 0.362) was present among studies. The pooled estimated success rate by the random-effects model was 90.1% (95% CI: 85.1% –93.5%; Figure 2). It's necessary to conduct subgroup analyses, due to different types of studies were eligible in this meta-analysis. For cohort studies, six studies enrolled 173 participants, and pooled estimated success rate by the random-effects model was 89.3% (95% CI: 81.3% –94.2%; Figure 3). In cross-sectional studies, four studies totaling 95 patients were included. Based on our analysis, the pooled estimate of success rate was 90.4%, and the 95% CI was 85.5% to 93.7%.

### HbA1c level

As a marker of chronic hyperglycemia, glycated hemoglobin (HbA1c) has now been used to diagnosis of diabetes and monitor glycemic control. The American Diabetes Association (ADA) and other major diabetes organizations also incorporated HbA1c into clinical practice guidelines, setting an HbA1c level of ≥ 6.5% (48 mmol/mol) as the cutoff value for the diabetes control [21, 22].

**Figure 4:**
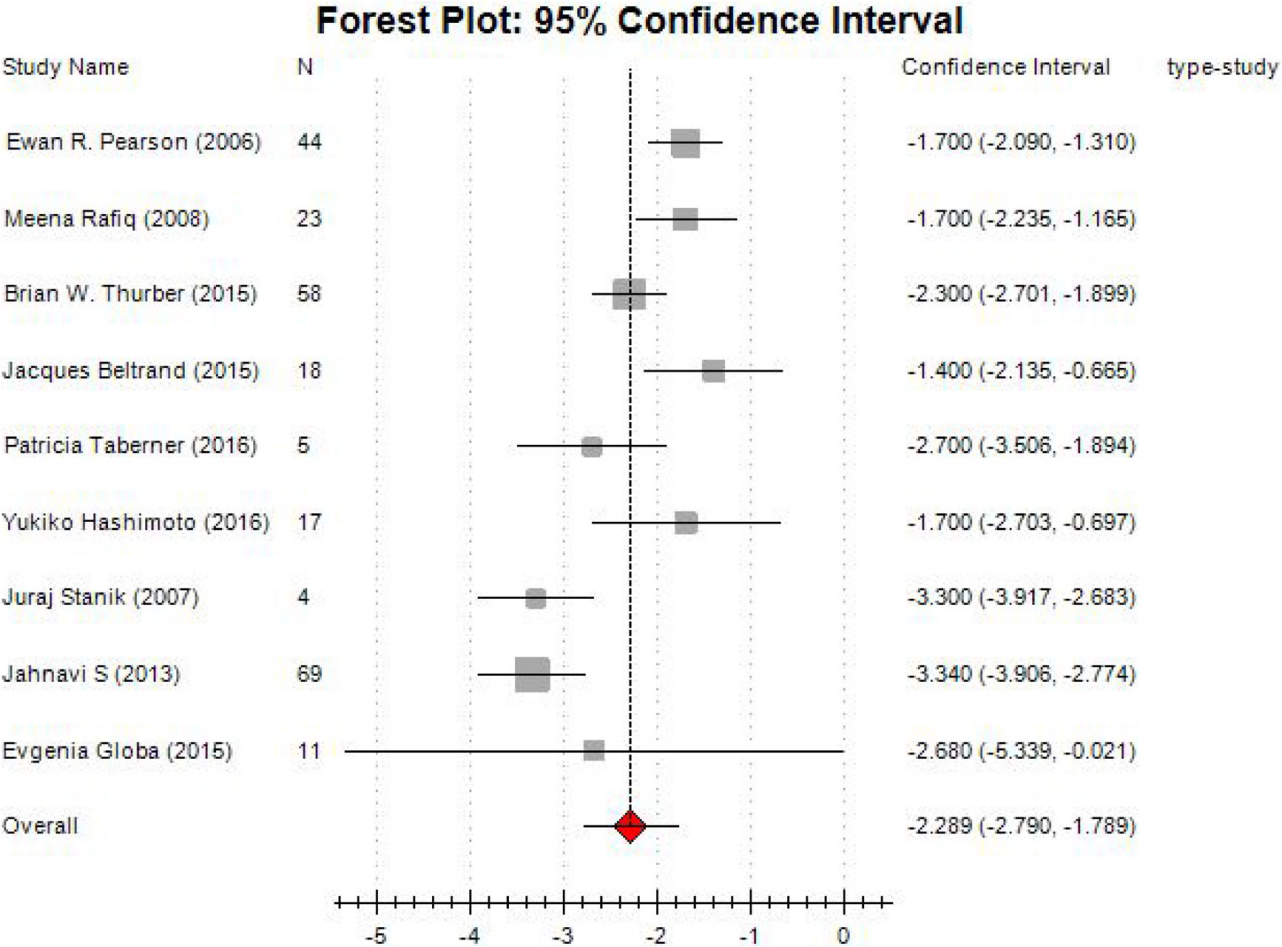
Forest plot of meta-analysis on changes of HbA1c level

In this systematic review, nine studies involving 268 participants provided data on HbA1c level. Compared with before treatment, HbA1c level was obviously decreased when sulfonylurea was administrated for the subjects. The test for heterogeneity of 9 studies demonstrated no heterogeneity (*P* = 0.00; I^2^ = 8.21%), and the random-effects model was performed. The pooled estimate of mean deviation, (MD) was –2.289, and the 95% CI was –2.790 to –1.789 (*P* < 0.001) (Figure 4).

**Figure 5:**
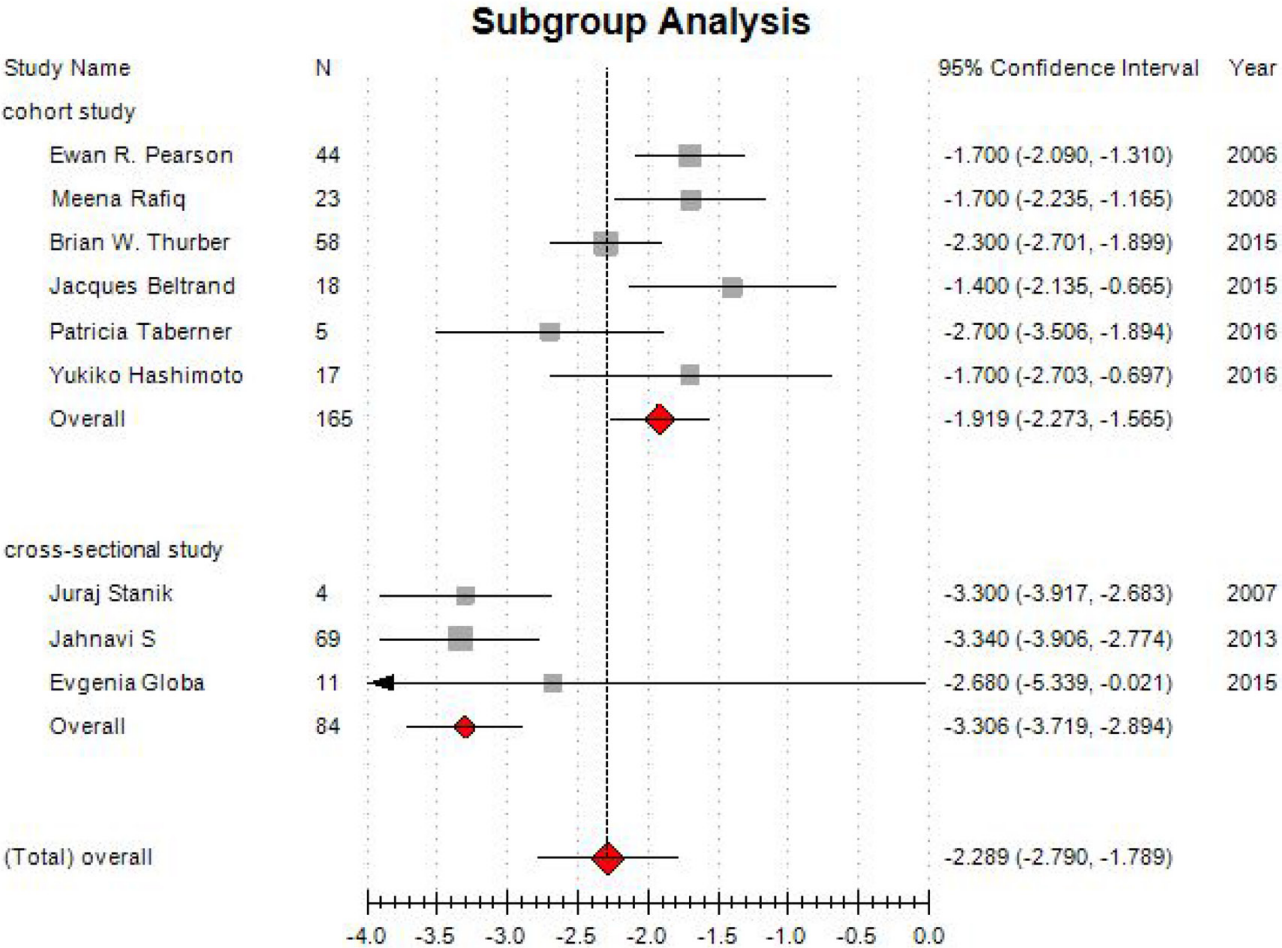
Forest plot of subgroup analysis on changes of HbA1c level

The results of subgroup analysis showed the combined MD in cohort studies was –1.919 (95% CI: –2.273~–1.565). For cross-sectional studies, the combined effect size was –3.306 (95% CI: –3.719 ~–2.894; Figure 5).

The results suggested that sulfonylurea are effective when used in NDM with K_ATP_-channel mutations.

### C-peptide levels

C-peptide in plasma may be a better measure of portal insulin secretion than insulin itself. In this systematic review, only three studies [12, 15, 20] mentioned to this indicator. It is elevated significantly after sulfonylurea treatment in these studies.

### Side effects

Two cohort studies [13, 14] and one cross-sectional study [20] reported adverse events. The most common side effect of sulfonylurea was the gastrointestinal reaction. In total, six patients had diarrhea, two associated with abdominal pain and one mild loss of appetite caused transitory weight loss. In addition, one patient had morning nausea, and one severe hypoglycemic episode was reported in a patient. All these side effects are transitory and resolved without discontinuing treatment.

### Publication bias

**Figure 6:**
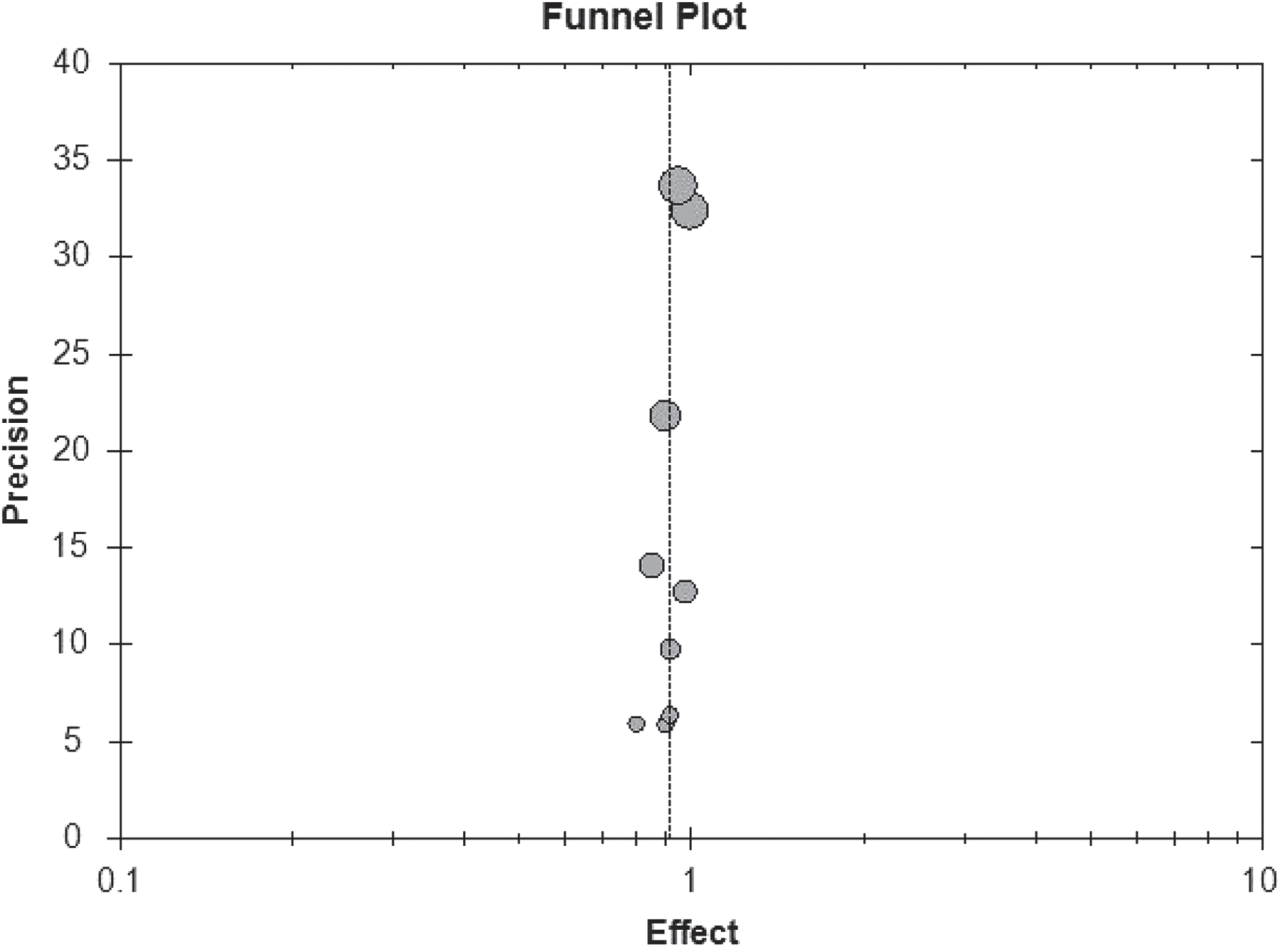
Funnel plot of meta-analysis on treatment success rate

**Figure 7:**
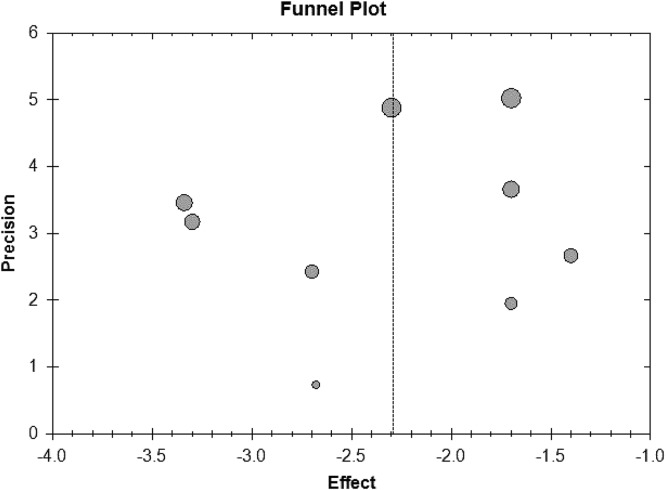
Funnel plot of meta-analysis on changes of HbA1c level

For the meta-analysis of sulfonylurea on treatment success rate and changes of HbA1c level, there were no evidence of significant publication bias by inspection of the funnel plot (Figure 6 and Figure 7).

## DISCUSSION

### Main findings

This systematic review and meta-analysis identified 6 cohort studies and 4 cross-sectional studies investigating the effect of sulfonylurea for the treatment of neonatal diabetes owing to K_ATP_-channel mutations. The meta-analysis revealed that sulfonylurea has a highly successful rate for treatment NDM due to heterozygous mutations of the genes KCNJ11 andABCC8 encoding the two subunits (Kir6.2 and SUR1) of K_ATP_ channel. Moreover, the finding was consistent in subgroup analyses for both in cohort studies and in cross-sectional studies. Furthermore, in some studies, sulfonylurea therapy is also safe and successful in neonatal diabetes patients before genetic testing results [23]. It is might be K_ATP_-channel mutations accounting for larger proportions of NDM. This implies that sulfonylurea might be used in all new diagnosed NDM; however, larger numbers of cases must be studied.

Blood glucose monitoring and adjustment of the treatment regimen is critically important in the management of diabetes. The way of conventional blood sugar monitoring is susceptible to short-term changes in diet, the presence of stress as well as other confounding factors [24]. In addition, determination of fasting plasma glucose has the poor compliance due to overnight fast. HbA1c could reflect average glycaemia over the past two to three months, owing to the lifespan of red blood cells is approximately 120 days [25]. Furthermore, advantages of HbA1c include the lack of participant preparation; high within-person reliability; and excellent standardization of the assay in most countries [26]. Besides, HbA1c is more reflective of macro-and microvascular complications than glucose [27]. Thus, HbA1c has become the gold standard for monitoring glycemic control in diabetes mellitus with the endorsement of influential diabetes societies and the World Health Organization.

In this systematic review, HbA1c had a significantly lower compared with before treatment. The pooled estimate of MD was –2.289, and the 95% CI was –2.790 to –1.789 (*P* < 0.001). The subgroup analysis showed a similar result for cohort studies and in cross-sectional studies.

In new diagnosed diabetic patients, it is vital to evaluate insulin secretion function of β cell. Also, residual insulin secretion has been proposed as a mean of classifying diabetes. However, insulin has a short half-life of a few minutes [28]. In addition, in patients with type I diabetes need to receive exogenous insulin, it also be difficult to estimate own insulin secretion by measuring insulin in serum.

Connecting peptide (C-peptide) is known for several decades. As a 31 amino acid segment, it is released in equimolar amounts together with insulin from the pancreatic beta cells [28]. Thus, C-peptide will reflect insulin secretion. Furthermore, C-peptide passes liver, has a longer half-life of half an hour, and is finally catabolized by the kidneys, and some is secreted in the urine [29]. Therefore, determination of C-peptide has been a common way of trying to standardize the evaluation of beta cell function. In this study, C-peptide level is obviously increased after sulfonylurea treatment in the three studies.

The common side effects are gastrointestinal reaction when sulfonylurea were used for NDM. In addition, nausea and hypoglycemic episode were also reported in a very small size of patient. As previously reported [30], all these side effects are transitory and resolved without discontinuing treatment. No other fatal side effects were reported.

These results indicated that sulfonylurea is a better choice for NDM with K_ATP_-channel mutations.

### Comparison with other studies

To the best of our knowledge, this meta-analysis is a first systematically and quantitatively evaluates the roles of sulfonylurea for NDM. However, all the included studies in this systematic review are observational study. Until now, there is no RCT studies to support its use. Further well-conducted trials that examine long-term outcomes are required.

### Implications for clinical practice

Though sulfonylurea for treatment neonatal diabetes is supported by some researchers and clinicians, insulin treatment is still acutely required in most infants with newly diagnosed diabetes mellitus to treat or prevent ketoacidosis and dehydration. In addition, sulfonylureas are not approved for use in infants in most countries. All these explanations limit the use of sulfonylureas for NDM especially for K_ATP_-channel mutations patients. Based on the results of our meta-analysis, sulfonylurea has a highly successful rate for treatment NDM and it also fulfill an evident glycemic control with HbA1c level significantly lower. Therefore, this meta-analysis provides the latest and most convincing references for developing clinical practice guidelines of NDM.

### Strengths and limitations

There are several strengths for our meta-analysis. Firstly, this meta-analysis was in compliance with the PRISMA guidelines and the recommendations of the Cochrane Collaboration. Secondly, we conducted this meta-analysis by exhaustive search without any restrictions. In addition, we performed several subgroup analyses to explore the potential sources of heterogeneity and increase the robustness of this meta-analysis.

Several limitations should be taken into consideration when interpreting the present results. First of all, all the included studies in our meta-analysis were observational studies. Observational studies are highly subject to selection bias and confounding which can contribute to underestimates or overestimates of the actual effect of an intervention [31]. Second, other factors such as spontaneous recovery tendency, time factor, environmental change were also the potential bias resulting from all the comparisons are self-control. It is reported that younger age at the time of initiation of sulfonylureas therapy is correlated with lower required doses of sulfonylureas therapy, shorter transition time and decreased likelihood of requiring additional diabetes medications [16]. In this meta-analysis, we did not

Besides, the sample sizes in this meta-analysis were not large and unpublished studies were not included in the analysis.

## CONCLUSIONS

The present systematic review and meta-analysis suggested that sulfonylurea had a highly successful rate for treatment NDM due to K_ATP_ channel mutations. Furthermore, sulfonylurea significantly decreased HbA1c level when compared with before treatment. In addition, sulfonylurea displayed sound safety except the mild gastrointestinal reaction.

## MATERIALS AND METHODS

### Selection criteria

Studies meeting the following criteria were included: (1) population: neonatal diabetes receiving sulfonylurea; (2) intervention: sulfonylurea with or without concurrent insulin; (3) comparison: insulin, before and latter intervention or non-intervention; (4) outcome: success rates of treatment, change of HbA1c and C-peptide; (5) design: all types of clinical studies (i.e., RCTs, cohort studies, case control studies, case series studies and case reports) which involved sulfonylurea for treatment neonatal diabetes were included.

### Search strategy

Pubmed, Embase and the Cochrane Library, were searched for studies reporting the sulfonylurea on the treatment of neonatal diabetes. All the data were searched from inception of the database to Jan. 2017. Search terms included those related to neonatal diabetes, sulfonylurea, glibenclamide and their variants. The search strategy for Pubmed and Embase were shown in Table 5. No language restriction was imposed. The reference lists of all retrieved articles were also reviewed to identify additional articles missed by using these search terms. In addition, we also manually checked the bibliographies of previous reviews and included trials to identify other potentially eligible trials.

**Table 5:** AHRQ quality assessment of included cross-sectional studies

	Study	Juraj Stanik 2007	Jahnavi S 2013	David Carmody 2014	Evgenia Globa 2015
Items	1	YES	YES	YES	YES
	2	YES	YES	YES	YES
	3	YES	UNCLEAR	UNCLEAR	YES
	4	YES	YES	YES	YES
	5	UNCLEAR	UNCLEAR	UNCLEAR	UNCLEAR
	6	YES	YES	YES	YES
	7	YES	NO	UNCLEAR	UNCLEAR
	8	UNCLEAR	UNCLEAR	UNCLEAR	UNCLEAR
	9	UNCLEAR	UNCLEAR	UNCLEAR	UNCLEAR
	10	UNCLEAR	YES	YES	YES
	11	YES	NO	UNCLEAR	UNCLEAR
**Quality Scores**		7	5	5	6

### Selection of studies and data extraction

Two authors (Zhang and Huang) independently carried out the initial search, deleted duplicate records, screened the titles and abstracts of every record. Full-text articles were obtained when information given in the title or abstracts either conformed to the selection criteria, or could not be ascertained owing to limited information. To include studies, data were extracted independently by the two authors (Zhang and Huang) and entered into a standardized Excel form. The following information was extracted from each study: first author, year of publication, country, study design, patient characteristics, number of patients enrolled, intervention, and outcome data. Any discrepancy was resolved by discussion and consensus.

### Risk of bias assessment

Two reviewers (Huang and Zhong) independently evaluated the methodological quality of identified studies. Newcastle-Ottawa Scale (NOS) was used for cohort studies and case control studies [32]. The NOS is a nine-star rating system designed for non-randomized studies, especially case-control and cohort studies. It contains 3 domains and 8 items. A maximum of 2 stars can be allotted in the item of comparability. The other items get a single star if appropriate methods have been reported.

The methodological quality of the cross-sectional studies was assessed using an 11-item checklist which was recommended by Agency for Healthcare Research and Quality (AHRQ) [33]. An item would be scored ‘0’ if it was answered ‘NO’ or ‘UNCLEAR’; if it was answered ‘YES’, then the item scored ‘1’. Article quality was assessed as follows: low quality = 0–3; moderate quality = 4–7; high quality = 8–11.

### Statistical method

Data were analyzed using the Open Meta-Analyst Beta 3.13 software (Tufts Medical Center, Boston, MA, USA). The indicators of rates such as success rate of treatment were analyzed by applying MetaAnalyst with the random-effects mode. For continuous outcome measurements, such as mean reduction value of HbA1c level and C-peptide, mean and standard deviation values (SD) were calculated and transformed if not given directly in the paper.

Heterogeneity among the included studies was evaluated by the *I^2^* test. A value greater than 50% to indicate substantial heterogeneity and sought the potential sources of heterogeneity (clinical heterogeneity and methodological heterogeneity) [34]. If the results of the studies could not combine using meta-analysis (due to significant clinical heterogeneity and unconventional methods used in the analysis of studies), they were just only presented individually.

Finally, publication bias was evaluated by using funnel plots.
